# Future Fertility of Patients With No Embryo Transfer in Their First IVF Cycle Attempts

**DOI:** 10.3389/fendo.2022.893506

**Published:** 2022-07-27

**Authors:** Xuli Zhu, Mingya Cao, Zhaohui Yao, Peiyang Lu, Yueming Xu, Guimin Hao, Zhiming Zhao

**Affiliations:** Department of Reproduction Medicine, The Second Hospital of Hebei Medical University, Shijiazhuang, China

**Keywords:** *in vitro* fertilization, no embryo transfer, cycle cancellation rate, poor ovarian response, cumulative live birth rate

## Abstract

**Objective:**

We aimed to evaluate the future outcomes of patients undergoing their first IVF (*in vitro* fertilization) attempt with no oocyte retrieved, no normal zygotes formed, or no embryos available for transfer and to identify factors affecting the live birth rate.

**Methods:**

Patients who underwent no transplantable embryo in their first IVF cycles but carried out several consecutive cycles between January 2012 to December 2020 were retrospectively enrolled and divided into three groups:group A (no egg retrieval), group B (no normal zygotes formed), and group C (no embryos available to transfer). The patients were also divided into the live birth group and non-live birth group according to whether they got a live baby or not. The clinical data and the cumulative clinical outcomes of groups were compared.

**Results:**

496 patients met the inclusion criteria and enrolled, with 121 patients with no oocytes retrieved in group A, 138 patients with no normal zygotes formed in group B, and 237 patients with no embryos available to transfer in group C. The age [(34.75(5.82) *vs* 31.91(5.31), *P*<0.001; 34.75(5.82) *vs* 32.25(5.72), *P*<0.001)] and baseline FSH level [(13.04(8.82) *vs* 10.52(7.39), *P*=0.005; 13.04(8.82) *vs* 9.91(5.95), *P*<0.001)] of women in group A were significantly higher than those in groups B and C. The stable cumulative live birth rate/patient of three groups achieved 18.18% (after 5 cycles, group A), 28.98% (after 3 cycles, group B) and 20.25% (after 7 cycles, group C). Moreover, the multivariate regression analysis showed that female age and basic FSH were main factors affecting live birth outcome of patients with no embryo transfer in their first IVF cycle attempts.

**Conclusions:**

The future clinical outcome may be better in women with no normal zygotes than those with no oocyte retrieved or no available embryo at their first IVF cycle attempts. The main factors influencing the live birth are age and ovarian reserve.

## Introduction

Embryo transfer is a key step for successful pregnancy of women through assisted reproduction, but this process may face cycle cancellation because of no oocyte retrieved, no normal zygote formed, or no available embryos. Considering the inherent poor outcomes, most studies have excluded patients with cycle cancellation at the beginning of research (1–3). The ESPART study reported the prevalence of cycle cancellation was 4.7% in poor responders (4), and another survey showed the risk of cycle cancellation caused by poor ovarian response was about 20% (5). However, the prevalence in the general population receiving *in vitro* fertilization (IVF) is obscure. A French study examined medical factors associated with early cessation of IVF in 5135 couples and found that couples who did undergo no embryo transfer during the first IVF cycle attempt were more likely to stop treatment early (6). Other studies (7–10) have found that the psychological burden of failure in non-pregnancy treatment is the reason for withdrawing from further treatment. There are currently few reports on the clinical outcome of follow-up treatment in these patients, but it is necessary to provide these patients with information about the final clinical outcome in all institutions carrying out the artificial reproduction technology (ART).

Based on the above observational studies and to answer the consultation of patients with no transplantable embryo in their first IVF cycle attempt, this study was performed to investigate the outcome of future fertility of patients undergoing their first IVF cycle attempt with no embryos transplanted as well as to identify factors that might affect the possibility to get a baby in subsequent IVF cycle attempt.

## Materials and Methods

### Subject

**Figure 1:**
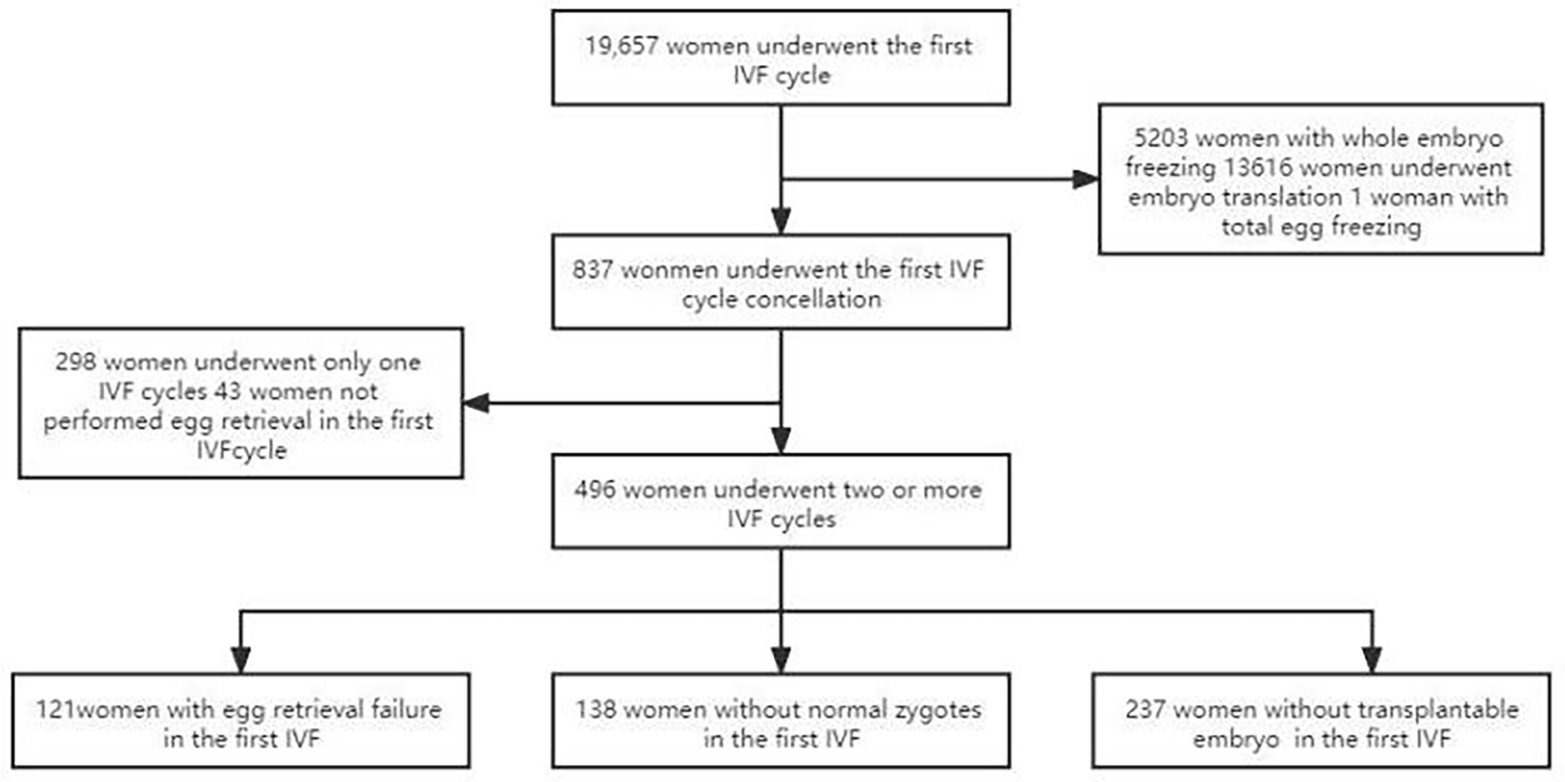
Flowchart of patient selection. A total of 19,657 couples underwent IVF cycle with oocytes to pick up, and 837 patients had cycle cancellation in their first IVF cycle attempts. 298 women underwent only one IVF cycle, of which 50 women were with no egg retrieved, 48 women with no normal zygotes formed and 200 women with the cancellation cycle due to poor embryo quality. 496 women met the study inclusion criteria and were enrolled.

The retrospective study was performed in consecutive women attending the IVF procedure in our hospital from January 2012 to December 2020. Inclusion criteria were women who wanted more cycle attempts after their first IVF cycles had been cancelled for some reasons even if they had reached the oocyte pick-up (OPU) stage and had undergone egg retrieval in their first IVF cycle. The first delivery was used as the end point of the study. Exclusion criteria include: (i) chromosomal abnormalities in either their spouse or pre-implantation genetic testing cycles; (ii) patients who did not continue the IVF cycle after the first cancelled cycle; (iii) patients who had no clinical outcome at the end of follow-up. This study was approved by the ethics committee of our hospital, and all patients had signed the informed consent to participate (**Figure 1**).

### Treatment Protocol and Pregnancy Criteria

The stimulation protocol was performed according to our center’s guidelines and was determined by the treating clinician based on individualized conditions. The stimulation protocol in fresh cycles included GnRH-a long protocol (luteal phase short-acting GnRH-a long protocol), short-acting GnRH-a protocol, GnRH-ant protocol (GnRH antagonist protocol), EFLL protocol (early-follicular phase long-acting GnRH agonist long protocol), PPOS protocol (progestin-primed ovarian stimulation protocol), and milder ovarian stimulation protocol. Different protocles may be used in the same person in different cycles.

Follicle growth and hormone levels were continuously monitored by ultrasound and blood tests. Human chorionic gonadotropin (hCG) was injected when the largest follicle diameter was bigger than 18 mm or the diameter of at least three follicles was bigger than 17 mm during the fresh cycle, and the oocytes were collected 36-38h after hCG injection. The oocytes were inseminated through conventional IVF/ICSI (intracytoplasmic sperm injection), and fertilization was observed 16-18h after insemination. Seventy-two hours after oocyte retrieval, whether to transplant or to freeze the embryo was based on embryo grading and the individual clinical situation of each patient. Blood β-hCG (+) was measured 14d after transfer as biochemical pregnancy, and clinical pregnancy was determined by ultrasonography at 28-30d when a gestational sac and primordial ventricular pulsation were observed.

### Embryo Score and Cycle Outcome Definition

The ASEBIR scoring criteria was used to assess the embryo at the cleavage stage (11). Embryos were classified into grades I-IV according to the number of blastomeres on day 3, proportion of fragmentation, uniformity of blastomeres, multinucleation, number of vacuoles, and normalness of the zona pellucida. The criteria for good quality embryos were 2PN (2 pronucleus) origin, 7-9 cells, fragmentation <10%, and basic homogeneity of the cleavage. Non-transferable embryos were defined as embryos with grade IV with no fused embryos formed in further culture at Day3; non-insemination was defined as no MII (mature) eggs 2 hours after degranulation; non-fertilization was defined as no normal progenitor nuclei observed after IVF fertilization with no oocyte cleavage. Group B (no normal zygote formation group) included non-fertilization and no normal progenitor nuclei.

### Grouping and Indicators

Patients were divided into three groups depending on the cause for the cancellation in their first IVF cycle: group A (no egg retrieval group), group B (no normal zygote formation group) including patients with non-insemination and non-fertilization, and group C (no embryos available to transfer). According to whether they had got a live baby or not, the patients were divided into live birth and non-live birth groups. The patients’ age, BMI (Body mass index), infertility years, infertility type, infertility factors (male or female), basic FSH or LH level, Gn (gonadotropin) dose and days, cumulative clinical pregnancy rate, and cumulative live birth rate of the three groups were compared. The Gn days, Gn dose, mean Oocytes/opu, total oocytes retrieved, average number of 2PN fertilization/opu, average number of transferred embryos/opu, cumulative clinical pregnancy rate/opu, cumulative clinical pregnancy rate/patient, https://fanyi.baidu.com/translate?aldtype=16047&query=%E7%AC%AC%E4%B8%80%E5%91%A8%E6%9C%9F%E6%82%A3%E8%80%85%E7%89%B9%E5%BE%81&keyfrom=baidu&smartresult=dict&lang=auto2zh - ##cumulative live birth rate/opu, https://fanyi.baidu.com/translate?aldtype=16047&query=%E7%AC%AC%E4%B8%80%E5%91%A8%E6%9C%9F%E6%82%A3%E8%80%85%E7%89%B9%E5%BE%81&keyfrom=baidu&smartresult=dict&lang=auto2zh - ##cumulative live birth rate/patient were recorded, respectively, among three groups according to the cycle rank, and the basic characteristics including age, BMI, basic FSH or LH level, Gn dose and days between the live birth and non-live birth groups in the first cycle were investigated.

### Statistical Analysis

The SPSS23.0 software (IBM, Chicago, IL, USA) was used for statistical analysis. Continuous variables were expressed as mean (standard deviation or SD) and categorical variable data were expressed as numbers and percentages. One-Way ANOVA was used to analyze the mean difference among groups, the Chi squre test was used to compare the classified data between groups, and the binary Logistic regression (Enter) was used to calculate the OR (odds ratio) value. The statistically significant difference was set at *P*<0.05.

## Results

### Subject

**Figure 2:**
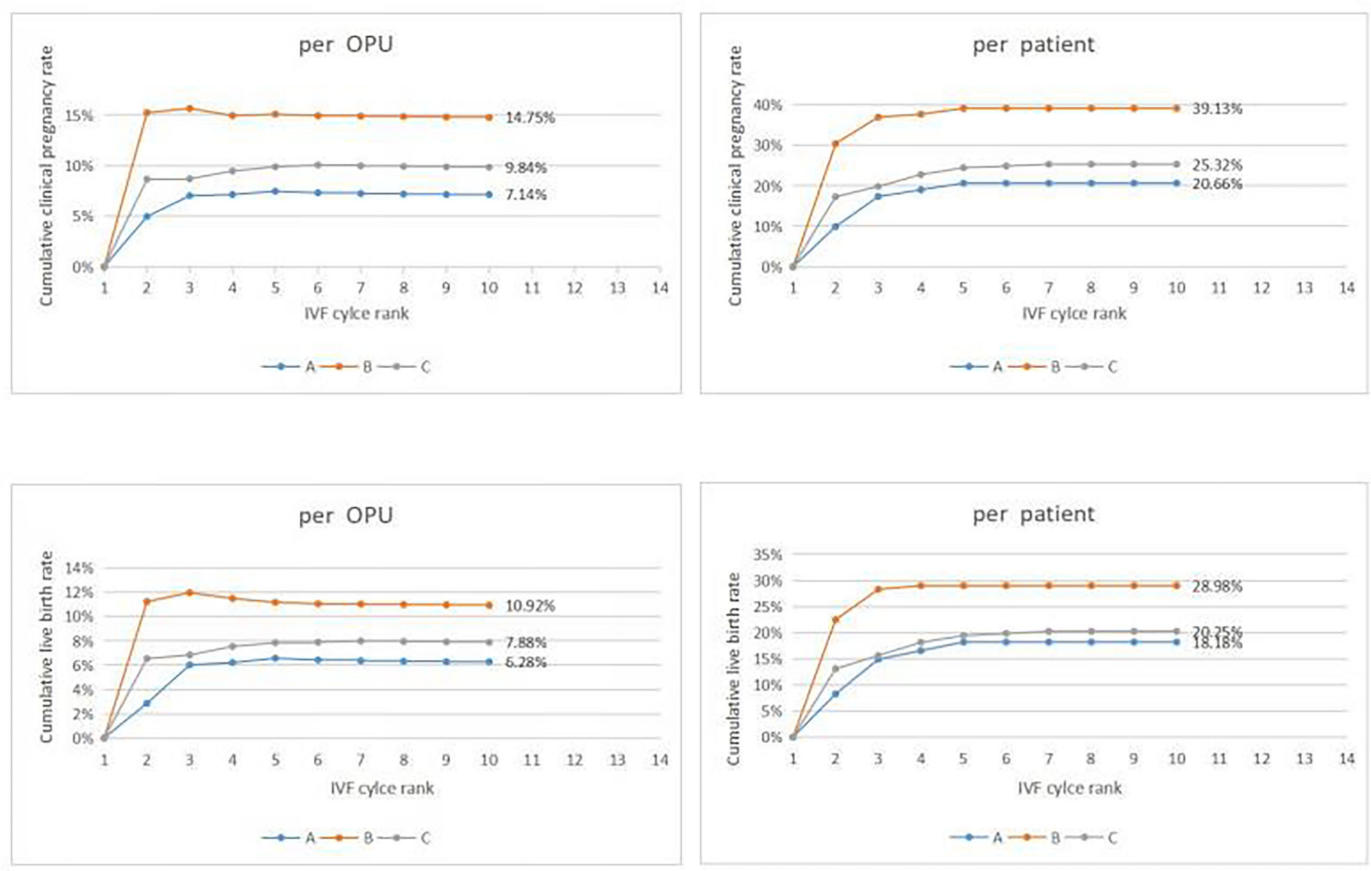
Cumulative clinical outcomes at cycle rank. In group A, The cumulative clinical pregnancy rates were 7.14% per OPU and 20.66% per patient, and the cumulative live birth rates were 6.28% per OPU and 18.18% per patient, with both rates reaching a plateau at the 5th OPU cycle. The cumulative live birth rate/patient in group B was 28.98%, which did not increase any more after cycle 3, whereas the cumulative live birth rates/patient in group C was 20.25% and reached a plateau after cycle 7.

During the study period, a total of 19,657 couples underwent the first IVF cycle and with oocytes being picked up, and 837 patients had cycle cancellation in their first IVF cycle attempts, resulting in a cancellation rate of 4.26% (837/19657). Among the 837 patients with cycle cancellation, 496 women who met the study inclusion criteria, with no embryo transfer, were enrolled to attend more cycles attempts. Among them, 121 cases of egg retrieval failure were assigned to group A, 138 cases who failed to form normal zygotes (including 83 cases of non-fertilization, 21 cases of abnormal fertilization, 33 cases of non-insemination, 1 case of non-cleavage) were assigned group B, and 237 without available embryo were assigned to group C. Baseline clinical characteristics and clinical outcomes of the three groups in the first IVF cycle are summarized in **Table 1**. Characteristics of the subsequent IVF cycles and the final reproductive outcomes stratified according to the IVF cycle rank between three groups are presented in **Tables 2.1**–**2.3** and **Figure 2**. The characteristics of live birth and non-live birth groups are showed in **Table 3**, and the outcomes of multivariate regression analysis between the two groups are demonstrated in **Table 4**.

**Table 1:** Comparison of baseline values and final reproductive outcomes in the first IVF cycle among the three groups.

groups	Group A	P1(A*vs*B)	Group B	P2(A*vs*C)	Group C	P3(B*vs*C)
Characteristics of patients in the first cycle						
Number of patients	121		138		237	
Age (years)	34.75(5.82)	<0.001	31.91(5.31)	<0.001	32.25(5.72)	ns
BMI (kg/m2)	23.41(5.18)	ns	23.70(3.71)	ns	23.4(3.51)	ns
FSH/mIU	13.04(8.82)	0.005	10.52(7.39)	<0.001	9.91(5.95)	ns
LH/mIU	5.81(5.75)	ns	5.14(4.03)	ns	7.93(46.97)	ns
AMH (ng/ml)	0.50 (0.56)	ns	0.68 (0.65)	0.044	1.15 (1.53)	0.002
Infertility years	4.42 (3.97)	ns	4.21 (3.33)	ns	4.29 (3.45)	ns
Infertility type						
Primary infertility	61/121	ns	91/138	<0.001	132/237	ns
Secondary infertility	60/121	0.011	47/138	ns	105/237	ns
Infertility factors						
Male (%)	16/121 (13.22%)	ns	21/138 (15.22%)	ns	33/237 (13.92%)	ns
Tubal (%)	58/121 (47.93%)	ns	57/138 (41.30%)	ns	101/237 (42.62%)	ns
Endometriosis (%)	7/121 (5.79%)	ns	6/138 (4.35%)	ns	15/237 (6.33%)	ns
Unexplained infertility (%)	5/121 (4.13%)	ns	13/138 (9.42%)	ns	20/237 (8.44%)	ns
Uterine factor (%)	2/121 (1.65%)	ns	3/138 (2.17%)	ns	0/237	ns
Ovulatory disorder (%)	25/121 (20.66%)	ns	28/138 (20.29%)	ns	46/237 (19.41%)	ns
Others (%)	4/121 (3.31%)	ns	46/138 (33.33%)	ns	4/237 (1.69%)	ns
https://fanyi.baidu.com/translate?aldtype=16047&query=%E7%AC%AC%E4%B8%80%E5%91%A8%E6%9C%9F%E6%82%A3%E8%80%85%E7%89%B9%E5%BE%81&keyfrom=baidu&smartresult=dict&lang=auto2zh - ##Treatment characteristics						
Ovarian stimulation protocol						
GnRH-a long protocol	15/121	<0.001	46/138	ns	70/237	<0.001
EFLL protocol	7/121	ns	17/138	ns	41/237	0.005
GnRH-ant protocol	30/121	ns	22/138	0.001	72/237	ns
Others	69/121	0.004	54/138	<0.001	54/237	<0.001
Gn days (days)	7.67(4.98)	0.001	9.28(3.30)	<0.001	9.93(3.68)	ns
Gn dose (IU)	2126.01(1474.67)	0.104	2367.66(1127.77)	<0.001	2622.98(1138.61)	0.063
Clinical outcome						
Number of OPU cycles	350		366		610	
Clinical pregnancy (n)	25		54		60	
Live birth(n)	22		40		48	
Cumulative clinical pregnancy/opu (%)	25/350(7.14%)	0.08	54/366(14.75%)	ns	60/610(9.84%)	0.021
Cumulative clinical pregnancy/patient (%)	25/121(20.66%)	0.001	54/138(39.13%)	ns	60/237(25.32%)	0.005
Cumulative live birth/opu (%)	22/350(6.28%)	0.027	40/366(10.92%)	ns	48/610(7.88%)	ns
Cumulative live birth/patient (%)	22/121(18.18%)	0.042	40/138(28.98%)	ns	48/237(20.25%)	0.054

Values are presented as number or mean (SD).

EFLL protocol, early-follicular phase long-acting GnRH agonist long protocol.

Others of ovarian stimulation protocol mean PPOS protocol, short-acting GnRH-a protocol, micro stimulation protocol, etc.ns, non-singnificant.

**Table 2.1:** IVF cycles’ characteristics and their final reproductive outcomes of group A stratified according to IVF cycle rank.

cycle rank	1	2	3	4	5	6	7	8	9	10
Gn days	7.57(4.90)	7.99(3.68)	9.04(4.86)	7.26(4.02)	7.36(3.64)	7.67(3.56)	5.00(2.65)	2.00(-)	5.50(2.12)	–
Gn dose	2100(1476)	2148(1184)	2545(1798)	1892(1345)	1932(1356)	1800(1168)	1225(943)	300(-)	1425(954)	–
No. of patients of opu with zero oocytes/total patients of opu	121/121	16/121	13/57	3/23	2/13	2/7	0/4	0/2	0/2	–
Mean Oocytes/opu	0	2.77 (3.64)	1.80(1.623)	1.96(1.64)	1.77(1.691)	1.00(0.816)	1.33(0.577)	1.00(0.00)	2.00(1.41)	–
Total of oocytes retrieved	0	316	101	45	23	7	4	2	4	–
Number Fertilizes oocytes of 2PN	0	166	55	25	12	2	2	1	1	–
Average number of 2PN fertilization/opu	0	2.07(2.74)	1.67(1.58)	1.56(1.55)	1.33(1.12)	0.67(0.58)	1.00(0.00)	1.00	1.00	–
Number of high quality embryos	0	43	17	7	2	1	0	0	0	–
Average number of high quality embryos/opu	0	0.57 (1.11)	0.55 (0.89)	0.44 (0.63)	0.29 (0.49)	0.33 (0.577)	–	–	–	–
Number of transferred embryos	0	53	25	8	5	2	1	1	1	–
Average number of transferred embryos/opu	0	1.77(0.73)	1.39(0.78)	1.60(0.894)	1.67(1.16)	1.00(0.00)	1	1	1	–
total patients of opu	121	121	57	23	13	7	4	2	2	0
https://fanyi.baidu.com/translate?aldtype=16047&query=%E7%AC%AC%E4%B8%80%E5%91%A8%E6%9C%9F%E6%82%A3%E8%80%85%E7%89%B9%E5%BE%81&keyfrom=baidu&smartresult=dict&lang=auto2zh - zh/en/javascript:void(0); https://fanyi.baidu.com/translate?aldtype=16047&query=%E7%AC%AC%E4%B8%80%E5%91%A8%E6%9C%9F%E6%82%A3%E8%80%85%E7%89%B9%E5%BE%81&keyfrom=baidu&smartresult=dict&lang=auto2zh - zh/en/javascript:void(0);Number of clinical pregnancies	0	12	9	2	2	0	0	0	0	0
Number of live births	0	10	8	2	2	0	0	0	0	0
Cumulative clinical pregnancy/opu (%)	0	12/242(4.96%)	21/299(7.02%)	23/322(7.14%)	25/335(7.46%)	25/342(7.31%)	25/346(7.23%)	25/348(7.18%)	25/350(7.14%)	25/350(7.14%)
Cumulative clinical pregnancy/patient(%)	0	12/121(9.92%)	21/121(17.36%)	23/121(19.01%)	25/121(20.66%)	25/121(20.66%)	25/121(20.66%)	25/121(20.66%)	25/121(20.66%)	25/121(20.66%)
https://fanyi.baidu.com/translate?aldtype=16047&query=%E7%AC%AC%E4%B8%80%E5%91%A8%E6%9C%9F%E6%82%A3%E8%80%85%E7%89%B9%E5%BE%81&keyfrom=baidu&smartresult=dict&lang=auto2zh - ##Cumulative live birth/opu(%)	0/121	10/242(2.86%)	18/299(6.02%)	20/322(6.21%)	22/335(6.57%)	22/342(6.43%)	22/346(6.36%)	22/348(6.32%)	22/350(6.28%)	22/350(6.28%)
https://fanyi.baidu.com/translate?aldtype=16047&query=%E7%AC%AC%E4%B8%80%E5%91%A8%E6%9C%9F%E6%82%A3%E8%80%85%E7%89%B9%E5%BE%81&keyfrom=baidu&smartresult=dict&lang=auto2zh - ##Cumulative live birth/patient(%)	0/121	10/121(8.26%)	18/121(14.88%)	20/121(16.53%)	22/121(18.18%)	22/121(18.18%)	22/121(18.18%)	22/121(18.18%)	22/121(18.18%)	22/121(18.18%)

Values are presented as number or mean (SD).

**Table 2.2:** IVF cycles’ characteristics and their final reproductive outcomes of group B stratified according to IVF cycle rank.

cycle rank	1	2	3	4	5	6	7	8	9	10
Gn days	9.28(3.30)	8.32(3.65)	8.91(3.56)	6.72(3.01)	8.14(2.08)	7.0(1.63)	8	2	8	12
Gn dose	2367(1127)	2242(1154)	2369(1194)	1633(979)	2121(716)	1650(851)	2400	300	1800	3600
Total of oocytes retrieved	571	560	162	55	22	6	4	2	2	3
Mean Oocytes/opu	4.17(5.70)	4.59(4.81)	3.31(3.57)	2.89(3.70)	2.44(2.92)	1.50(1.00)	2	1	2	3
Number Fertilizes oocytes of 2PN	0	173	56	15	14	2	2	2	2	3
Average number of 2PN fertilization/opu	0	3.20(3.87)	1.93(2.99)	1.36(1.12)	2.00(2.71)	0.67(0.577)	2	2	2	3
Number of high quality embryos	0	57	15	6	3	0	0	0	0	0
Average number of high quality embryos/opu	0	0.78(1.85)	0.60(1.96)	0.67(1.66)	0.60(0.89)	–	–	–	–	–
Number of transferred embryos	0	93	19	3	0	0	2	2	0	0
Average number of transferred embryos/opu	0	1.82(0.65)	1.73(0.65)	1.50(1.15)	–	–	2	2	–	–
Number of no normal zygotes/opu	138	7	3	0	1	1	0	0	0	0
total patients of opu	138	138	50	22	10	4	1	1	1	1
https://fanyi.baidu.com/translate?aldtype=16047&query=%E7%AC%AC%E4%B8%80%E5%91%A8%E6%9C%9F%E6%82%A3%E8%80%85%E7%89%B9%E5%BE%81&keyfrom=baidu&smartresult=dict&lang=auto2zh - ## https://fanyi.baidu.com/translate?aldtype=16047&query=%E7%AC%AC%E4%B8%80%E5%91%A8%E6%9C%9F%E6%82%A3%E8%80%85%E7%89%B9%E5%BE%81&keyfrom=baidu&smartresult=dict&lang=auto2zh - zh/en/javascript:void(0);Number of clinical pregnancies	0	42	9	1	2	0	0	0	0	0
https://fanyi.baidu.com/translate?aldtype=16047&query=%E7%AC%AC%E4%B8%80%E5%91%A8%E6%9C%9F%E6%82%A3%E8%80%85%E7%89%B9%E5%BE%81&keyfrom=baidu&smartresult=dict&lang=auto2zh - zh/en/javascript:void(0); https://fanyi.baidu.com/translate?aldtype=16047&query=%E7%AC%AC%E4%B8%80%E5%91%A8%E6%9C%9F%E6%82%A3%E8%80%85%E7%89%B9%E5%BE%81&keyfrom=baidu&smartresult=dict&lang=auto2zh - zh/en/javascript:void(0);Number of live births	0	31	8	1	0	0	0	0	0	0
Cumulative clinical pregnancy rate/opu(%)	0	42/276(15.22%)	51/326(15.64%)	52/348(14.94%)	54/358(15.08%)	54/362(14.92%)	54/363(14.88%)	54/364(14.84%)	54/365(14.79%)	54/366(14.75%)
Cumulative clinical pregnancy rate/patient(%)	0	42/138(30.43%)	51/138(36.96%)	52/138(37.68%)	54/138(39.13%)	54/138(39.13%)	54/138(39.13%)	54/138(39.13%)	54/138(39.13%)	54/138(39.13%)
https://fanyi.baidu.com/translate?aldtype=16047&query=%E7%AC%AC%E4%B8%80%E5%91%A8%E6%9C%9F%E6%82%A3%E8%80%85%E7%89%B9%E5%BE%81&keyfrom=baidu&smartresult=dict&lang=auto2zh - ##Cumulative live birth rate/opu(%)	0/138	31/276(11.23%)	39/326(11.96%)	40/348(11.49%)	40/358(11.17%)	40/362(11.05%)	40/363(11.02%)	40/364(10.99%)	40/365(10.96%)	40/366(10.92%)
https://fanyi.baidu.com/translate?aldtype=16047&query=%E7%AC%AC%E4%B8%80%E5%91%A8%E6%9C%9F%E6%82%A3%E8%80%85%E7%89%B9%E5%BE%81&keyfrom=baidu&smartresult=dict&lang=auto2zh - ##Cumulative live birth rate/patient(%)	0/138	31/138(22.46%)	39/138(28.26%)	40/138(28.98%)	40/138(28.98%)	40/138(28.98%)	40/138(28.98%)	40/138(28.98%)	40/138(28.98%)	40/138(28.98%)

Values are presented as number or mean (SD).

**Table 2.3:** IVF cycles’ characteristics and their final reproductive outcomes of group c stratified according to IVF cycle rank.

cycle rank	1	2	3	4	5	6	7	8	9	10
Gn days	9.97(3.63)	9.366(2.92)	8.25(3.80)	8.69(4.07)	8.06(3.51)	9.43(4.54)	5.00(4.24)	6.66(5.13)	7.00(9.90)	7.50(0.71)
Gn dose	2621(1136)	2427(983)	2150(1155)	2181(1366)	2107(1280)	1882(917)	1087(1007)	1300(1128)	1575(2227)	1687(159)
Total of oocytes retrieved	1079	1367	288	68	47	17	3	4	3	4
Mean Oocytes/opu	4.55(4.80)	5.84(5.62)	4.57(7.34)	2.34(2.73)	2.94(3.25)	1.89(1.36)	0.60(0.84)	1.33(0.57)	1	2
Number Fertilizes oocytes of 2PN	374	467	144	29	17	8	2	2	2	1
Average number of 2PN fertilization/opu	1.82(2.65)	3.16(3.50)	3.35(5.61)	1.53(1.17)	1.42(1.08)	1.14(0.37)	–	–	–	–
Number of high quality embryos	0	42	6	3	1	1	0	0	0	0
Average number of high quality embryos/opu	0	0.45(1.06)	0.26(0.54)	0.33(0.50)	0.14(0.38)	0.20(0.447)		0	–	
Number of transferred embryos	0	79	17	4	4	2	1	–	–	–
Average number of transferred embryos/transplantation cycle	0	1.68(0.63)	1.89(0.61)	2	2	2	–	–	–	–
No transplantable embryo/opu	237	38	11	3	3	3	2	2	1	2
total patients of opu	237	237	67	30	16	10	5	3	3	2
https://fanyi.baidu.com/translate?aldtype=16047&query=%E7%AC%AC%E4%B8%80%E5%91%A8%E6%9C%9F%E6%82%A3%E8%80%85%E7%89%B9%E5%BE%81&keyfrom=baidu&smartresult=dict&lang=auto2zh - zh/en/javascript:void(0); https://fanyi.baidu.com/translate?aldtype=16047&query=%E7%AC%AC%E4%B8%80%E5%91%A8%E6%9C%9F%E6%82%A3%E8%80%85%E7%89%B9%E5%BE%81&keyfrom=baidu&smartresult=dict&lang=auto2zh - zh/en/javascript:void(0);Number of clinical pregnancies	0	41	6	7	4	1	1	0	0	0
https://fanyi.baidu.com/translate?aldtype=16047&query=%E7%AC%AC%E4%B8%80%E5%91%A8%E6%9C%9F%E6%82%A3%E8%80%85%E7%89%B9%E5%BE%81&keyfrom=baidu&smartresult=dict&lang=auto2zh - ## https://fanyi.baidu.com/translate?aldtype=16047&query=%E7%AC%AC%E4%B8%80%E5%91%A8%E6%9C%9F%E6%82%A3%E8%80%85%E7%89%B9%E5%BE%81&keyfrom=baidu&smartresult=dict&lang=auto2zh - zh/en/javascript:void(0);Number of live births	0	31	6	6	3	1	1	0	0	0
Cumulative clinical pregnancy rate/opu(%)	0	41/474(8.65%)	47/541(8.69%)	54/571(9.46%)	58/587(9.88%)	59/587(10.05%)	60/602(9.97%)	60/605(9.92%)	60/608(9.87%)	60/610(9.84%)
Cumulative clinical pregnancy rate/patient(%)	0	41/237(17.29%)	47/237(19.83%)	54/237(22.78%)	58/237(24.47%)	59/237(24.89%)	60/237(25.32%)	60/237(25.32%)	60/237(25.32%)	60/237(25.32%)
https://fanyi.baidu.com/translate?aldtype=16047&query=%E7%AC%AC%E4%B8%80%E5%91%A8%E6%9C%9F%E6%82%A3%E8%80%85%E7%89%B9%E5%BE%81&keyfrom=baidu&smartresult=dict&lang=auto2zh - ##Cumulative live birth rate/opu(%)	0/237	31/474(6.54%)	37/541(6.84%)	43/571(7.54%)	46/587(7.85%)	47/597(7.89%)	48/602(7.99%)	48/605(7.95%)	48/608(7.91%)	48/610(7.88%)
https://fanyi.baidu.com/translate?aldtype=16047&query=%E7%AC%AC%E4%B8%80%E5%91%A8%E6%9C%9F%E6%82%A3%E8%80%85%E7%89%B9%E5%BE%81&keyfrom=baidu&smartresult=dict&lang=auto2zh - ##Cumulative live birth rate/patient(%)	0/237	31/237 13.08%	37/237 15.61%	43/237 18.14%	46/237 19.41%	47/237 19.83%	48/237 20.25%	48/237 20.25%	48/237 20.25%	48/237 20.25%

Values are presented as number or mean (SD).

**Table 3:** Basic characteristics of the first cycle in live birth group and non-live birth group.

	Live birth group	non live birth group	p
Proportion of patients			0.06
Group A	22	99	
Group B	40	98	
Group C	48	189	
Age (years)	31.21 (4.65)	33.22 (5.95)	0.001
BMI (kg/m2)	23.36 (3.55)	23.51 (4.14)	ns
FSH/mIU	9.08 (6.15)	11.41 (7.49)	0.004
LH/mIU	5.43 (4.05)	7.02 (3.72	ns
Infertility years	4.05 (3.24)	4.37 (3.62)	ns
AMH (ng/ml)	1.71 (2.91)	1.06 (1.18)	ns
Infertility type			ns
Primary infertility	65	219	
Secondary infertility	45	167	
Ovarian stimulation protocol			ns
GnRH-a long protocol	35	96	
EFLL protocol	20	45	
GnRH-ant protocol	21	103	
others	34	142	
Gn days (days)	10.05 (3.98)	8.99 (3.95)	0.01
Gn dose (IU)	2464 (1121)	2428 (1265)	ns

Values are presented as number or mean (SD).

EFLL protocol, early-follicular phase long-acting GnRH agonist long protocol.

Others of ovarian stimulation protocol mean PPOS protocol, short-acting GnRH-a protocol, micro stimulation protocol, etc.ns, non-singnificant.

**Table 4:** Multivariate regression analysis of IVF cycle between the live birth group and non-live birth group.

	Live birth group	non live birth group	*P*	OR value(95%CI)	*p*	OR adjusted value(95%CI)
Number of cycles	110	1216				
Cycle outcome type						
Not transplanted	0	785	–	–	–	–
Fresh cycle transplantation	65	227	–	–	–	–
https://fanyi.baidu.com/translate?aldtype=16047&query=%E7%AC%AC%E4%B8%80%E5%91%A8%E6%9C%9F%E6%82%A3%E8%80%85%E7%89%B9%E5%BE%81&keyfrom=baidu&smartresult=dict&lang=auto2zh - ##Thawed embryo transfer from Whole embryo freezing	45	204	–	–	–	–
Fertilization mode						
No insemination	0	217	–	–	–	–
IVF	65	664	–	–	–	–
ICSI	45	335	–	–	–	–
Patient first cycle type						
Type A	22	328		1		1
Type B	40	326	0.388	1.28(0.726,2.28)	0.189	0.438(0.128,1.500)
Type C	48	562	0.272	1.33(0.80,2.21)	0.091	3.174(0.884,5.345)
age	31.21(4.65)	33.22(5.95))	0.04	0.956 (0.916, 0.998)	0.042	0.935(0.876,0.997)
BMI	23.36(3.55)	23.51(4.14)	ns	0.984 (0.928, 1.043)	ns	1.030(0.965,1.100)
FSH	9.08(6.15)	11.41(7.49)	0.032	0.953 (0.911, 0.996)	0.046	0.937(0.878,0.999)
LH	5.43(4.05)	7.02(37.22)	ns	0.999 (0.994, 1.005)	ns	1.000(0.996,1.004)
Gn days	10.05(3.98)	8.99(3.95)	ns	1.067 (0.964, 1.181)	ns	1.073(0.984,1.171)
Gn dose	2464(1122)	2428(1265)	ns	1	ns	1

Values are presented as number or mean (SD), the OR (odds ratio) value and 95% CI (confidence interval), and OR adjusted value (95% CI) are obtained by adjusting the male factor, ovarian stimulation program and other confounding factors.ns, non-singnificant.

### Baseline and Characteristics Among Three Groups

A significant difference existed in female age, basic FSH level and Gn days between group A and group B (*P*< 0.001, *P*=0.005, *P*=0.001)as well as between groups A and C (*P*< 0.001, *P*< 0.001, *P*< 0.001). Cumulative clinical pregnancy rates per OPUand per patient in group B were 14.75% and 39.13%, respectively, which were better than those in other two groups. Cumulative live birth rates per OPU and per patient were 10.92% and 28.98%, respectively, in group B, which were significantly higher than those in group A (*P*=0.027, *P*=0.042), but not significantly (*P*>0.05)different from those in group C. Both cumulative pregnancy rate and cumulative live birth rates were not significantly (*P*>0.05) different between group A and C (**Table 1**).

### Cumulative Clinical Outcomes at Cycle Rank

In group A, the cumulative clinical pregnancy rates were 7.14% per OPU and 20.66% per patient, and the cumulative live birth rates were 6.28% per OPU and 18.18% per patient, all of which reached a plateau at the 5th OPU cycle (**Table 2.1** and **Figure 2**). The cumulative live birth rates/patient in group B was 28.98% and did not increase any more after cycle 3 (**Table 2.2** and **Figure 2**), whereas the cumulative live birth rates/patient in group C was 20.25% and reached a plateau after cycle 7 (**Table 2.3** and **Figure 2**).

### Comparison Between Live Birth and Non-Live Birth Groups and Factors Influencing Live Birth

Both age (31.21 ± 4.65 *vs* 33.22 ± 5.95, *P*=0.001) and basic FSH level (9.08 ± 6.15 *vs* 11.41 ± 7.49, *P*=0.004) in the live birth group were statistically signficantly lower than those in other groups (**Table 3**). The multivariate regression analysis showed that age and basic FSH level were predict factors for live birth (ORadjusted 0.935, 95% CI 0.876-0.997, *P*=0.042; ORadjusted 0.937, 95% CI 0.878-0.999, *P*=0.046) (**Table 4**).

## Discussion

This study evaluated the chance of a live birth in women with no embryo transfer at their first IVF cycle attempt over their subsequent IVF treatment course and factors affecting the live birth.

In our center, the total cycle cancellation rate was nearly 10%, and the cancellation rate of the first cycle was 4.9%. This may be related to embryo transfer strategies as well as the ovarian stimulation protocols used in our hospital. For example, the cycle cancellation rate of low ovarian response with minimal stimulation was 10% while that with long protocol was 2.9% (12). A meta-analysis in 2021 showed that the cycle cancellation rate of mild stimulation was higher than that of conventional ovarian stimulation in the low ovarian response group (13). Siristatidis et al (14) also found that the cycle cancellation rate of mild stimulation was significantly higher than that of long-term and antagonist regimens (36.4% *vs* 12%). A recent study showed that participants experienced an overall feeling of “loss and loneliness” when they had no experience of embryo transfer. The situation was described as a shock unprepared for them. Their need for relevant information has not been met (15). The results of the present study showed that some data could provide these patients with some information in terms of no embryo transfer in their first IVF cycle attempt.

In the group with no egg retrieved, the proportion of the patients in the first IVF cycle cancellation was 21.54% [(121 + 50)/(496 + 298)], and 22 of these women achieved a live birth in their subsequent cycles. Furthermore, there was no significant difference in the cycle source and the mode of fertilization through analysis of the original data. In patients with no eggs being retrieved in our study, the ovarian response was low, and their average age was higher than those in the other two groups. In the end, a 18% cumulative live birth rate was achieved in these patients after the 5th IVF cycle, which was similar to that of the study by Raoul Orvieto et al. (16). For these patients, the reproductive outcome will not change after the 5th IVF OPU cycle being tried, and the next ovulation stimulation cycle is suggested to be abandoned. Nonetheless, the patient’s own decision should always be respected.

The proportion of women with no normal zygotes formed in the first IVF cycle cancellation was 23.43% [(138 + 48)/(496 + 298)], and 40 of the 138 patients achieved a live birth eventually. In our hospital, when the number of eggs retrieved was less than or equal to 3, ICSI was not rescued. The majority of these patients had fewer eggs retrieved, which might be one reason for fertilization failure. Even though there was no significant difference in the fertilization methods (IVF *vs* ICSI) in the live birth cycle (9.46%*vs*13.27%), 54 cases were changed to ICSI after the first cycle of IVF, and 13 cases obtained a live birth, with the live birth rate of 24.07%. According to these outcomes, changing the mode of insemination in the second cycle may bring a better outcome. Some scholars (17) found that compared with the hCG single trigger group, the oocyte fertilization rate (73.1% *vs* 58.6%), clinical pregnancy rate (33% *vs* 20.7%), live birth rate (26.9% *vs* 14.5%), abortion rate (17.4% *vs* 37.0%), and embryo transfer elimination rate (6.1% *vs* 15.4%) in the double trigger group were significantly different. This may suggest that changing the trigger scheme, grasping the correct trigger time so as to get the target follicles as far as possible, and getting the MII (mature) eggs may improve the pregnancy outcomes. Furthermore, some patients may improve the clinical outcomes by using some new techniques like AOA (assisted oocyte activation), IVM (*in vitro* maturation), and testing for associated genes. A retrospective study (18) showed that AOA intervention after ICSI fertilization failure significantly increased the normal fertilization rate (52.1%), the cumulative clinical pregnancy rate (47.1%) and the live birth rate (29.4%).

The proportion of patients with the cancellation cycle caused by poor embryo quality in the first IVF cycle was 55.04% [(237 + 200)/(496 + 298)]. These patients are more challenging for the ART. In our study, these patients’ age was not high, they had more eggs, but the CLBR was low. In the ovulation induction of these patients, 25 cases with the antagonist regimen obtained a live birth, whereas 23 cases with other protocols did not. It seems that the antagonist regimen had a trend of increasing the live birth rate, but no significant difference was found in our study (10.25% *vs* 6.28%, P =0.07). Similarly, the fertilization mode of the live birth cycle was not signfiicantly different. Moreover, although no differences were found in various infertility factors among these patients in our study, a recent study (19) reported that AOA could improve the clinical pregnancy and live birth rate in patients with male factors (oligoasthenospermia [OAT]), advanced age, polycystic ovary syndrome (PCOS), and unexplained infertility. In current literature, blastocyst transfer after ICSI-AOA according to different infertility factors may help to improve the clinical outcome of these patients. Besides, shortening the embryo culture time to the first or second day and carrying out gamete transfer or zygote transfer may be a goodway to improve the reproductive outcome of patients with embryonic development block from the embryologists’ point of view.

Female age and ovarian reserve are still the main factors influencing the live birth of patients with first IVF cycle cancellation. However, clinical quality control, personalized treatment, or changes in ovulation induction and fertilization mode may help them achieve a live birth. Therefore, for patients with good financial resources, the findings of the present study suggest extending the number of OPU cycles up to the 3rd, 5th, or 7th cycle even if the reasons for cancellation of the first IVF cycle are different.

Some limitations existed in this study, including the retrospective and one-center design, a small cohort of patients, and Chinese patients enrolled only. Moreover, the reasons for patient cycle cancellation are multifarious and complex, and patients with non-fertilization, fertilization failure and abnormal fertilization might have been assigned to one group for analysis, which limits the ability to control for potential unknown confounding factors. All these factors may affect the generalization of the outcome of this study. Future studies will have to resolve all these issues for better outcomes.

In conclusion, future clinical outcomes may be better in women with no normal zygotes than those with no oocyte retrieved or no available embryo at their first IVF cycle attempts. The main factors influencing the live birth are age and ovarian reserve.

## Data Availability Statement

The raw data supporting the conclusions of this article will be made available by the authors without undue reservation.

## Ethics Statement

The studies involving human participants were reviewed and approved by Ethics Committee of The Second Hospital of Hebei Medical University. The patients/participants provided their written informed consent to participate in this study.

## Author Contributions

Study design: ZZ and GH. Data collection: XZ, MC, ZY, and PL. Data analysis: XZ and MC. Supervision: YX. Writing the original article: MC. Revision: ZZ. All authors contributed to the article and approved the submitted version.

## Funding

This study was supported by S&T Program of Hebei (22377742D), Natural Science Foundation of Hebei Province(Beijing-Tianjin-Hebei Cooperation Special Project) (H2019206712).

## Conflict of Interest

The authors declare that the research was conducted in the absence of any commercial or financial relationships that could be construed as a potential conflict of interest.

## Publisher’s Note

All claims expressed in this article are solely those of the authors and do not necessarily represent those of their affiliated organizations, or those of the publisher, the editors and the reviewers. Any product that may be evaluated in this article, or claim that may be made by its manufacturer, is not guaranteed or endorsed by the publisher.
